# Supplementary material for: Coupled Cluster Theory for Nonadiabatic Dynamics: Nuclear Gradients and Nonadiabatic Couplings in Similarity Constrained Coupled Cluster Theory
Source: J Chem Theory Comput. 2024 Aug 13;20(16):7080–92. doi: 10.1021/acs.jctc.4c00276 (PMC11360132; doi:10.1021/acs.jctc.4c00276)
Supplement: Supplementary file 1 — ct4c00276_si_001.pdf [file ct4c00276_si_001.pdf]

Supporting information for "Coupled cluster theory for nonadiabatic dynamics: nuclear gradients and nonadiabatic couplings in similarity constrained coupled cluster theory"

Eirik F. Kjønstad,[1,2,3,a)] Sara Angelico,[1] and Henrik Koch[1,b)]

[1)] *Department of Chemistry, Norwegian University of Science and Technology, 7491 Trondheim, Norway*

[2)] *Department of Chemistry, Stanford University, Stanford, CA, 94305, USA*

[3)] *Stanford PULSE Institute, SLAC National Accelerator Laboratory, Menlo Park, CA, 94025, USA*

(Dated: 21 May 2024)

[a)] Electronic mail: eirik.kjonstad@ntnu.no

[b)] Electronic mail: henrik.koch@ntnu.no

CONTENTS

S1. Stationarity equations: derivative coupling S3

S2. Orthogonality conditions and choice of metric S6

S3. One and two-electron density corrections S7

S4. Response vectors for excited state stationarity S14

S5. Response contributions from the orthogonality S17

S6. Comparison with numerical gradients S18

S7. Formaldimine MECIs S22

S8. Multiple solutions S23

A. Protonated formaldimine S23

B. Lithium hydride S24

S9. Thymine MECIs S26

S10. Coupling elements for lithium hydride S27

References S28

S1. STATIONARITY EQUATIONS: DERIVATIVE COUPLING

In the main text, we outline the derivation of the stationarity equations for the nuclear gradient. Here we outline the derivation for the derivative coupling elements. The coupling Lagrangian is given as

$$\begin{aligned}\mathscr{L}_{mn} = O_{mn} + \sum_{k\in\mathscr{K}} \bar{\boldsymbol{\mathcal{L}}}_k^{\mathrm{T}}(\bar{\boldsymbol{\mathcal{H}}} - E_k)\boldsymbol{\mathcal{R}}_k \\ + \sum_{k\in\mathscr{K}} \bar{E}_k(1 - \langle\Lambda_k|R_k\rangle) + \sum_{k\neq l\in\mathscr{K}} \bar{\gamma}_{kl}\mathscr{O}_{kl} + \sum_{\mu}\bar{\zeta}_\mu\Omega_\mu + \sum_{ai}\bar{\kappa}_{ai}F_{ai}.\end{aligned} \tag{1}$$

The Lagrangian is identical to $\mathscr{L}_n$, except for the first term, which is E_n for $\mathscr{L}_n$ and O_{mn} for $\mathscr{L}_{mn}$. This implies that

$$\frac{\partial\mathscr{L}_{mn}}{\partial\Lambda_\mu^k}\Big|_0 = 0 = R_\mu^k\big(- E_k\langle\bar{\mathcal{L}}_k|\mathcal{R}_k\rangle - \bar{E}_k\big) \tag{2}$$

and, hence,

$$\bar{E}_k = -E_k\langle\bar{\mathcal{L}}_k|\mathcal{R}_k\rangle. \tag{3}$$

Note that these multipliers differ by one term (which stems from E_n in $\mathscr{L}_n$) from the corresponding $\bar{E}_k$ for the gradient. Similarly to the gradient, this allows to recast the Lagrangian in the (sometimes) convenient form

$$\begin{aligned}\mathscr{L}_{mn} = O_{mn} + \sum_{k\in\mathscr{K}}(\langle K_k|\bar{H}|R_k\rangle + \bar{L}_0^k\langle\mathrm{HF}|\bar{H}|\mathcal{R}_k\rangle) \\ + \langle\bar{\zeta}|\bar{H}|\mathrm{HF}\rangle + \sum_{ai}\bar{\kappa}_{ai}F_{ai} \\ + \sum_{k\in\mathscr{K}}\bar{E}_k(1 - \langle\Lambda_k|R_k\rangle) + \sum_{k\neq l\in\mathscr{K}}\bar{\gamma}_{kl}\mathscr{O}_{kl}\end{aligned} \tag{4}$$

with

$$\langle K_k| = -\langle\bar{\mathcal{L}}_k|\mathcal{R}_k\rangle\langle\Lambda_k| + \langle\bar{L}_k|. \tag{5}$$

Like for the gradient, we assume that $\langle\Lambda_k| = \langle L_k|$.

Stationarity with respect to R_0^k gives the same result as for the gradient:

$$\frac{\partial\mathscr{L}_{mn}}{\partial R_0^k}\Big|_0 = \langle\mathcal{L}_m|\mathrm{HF}\rangle\delta_{kn} - \omega_k\bar{L}_k^0 + \sum_{l\neq j\in\mathscr{K}}\bar{\gamma}_{lj}\frac{\partial\mathscr{O}_{lj}}{\partial R_0^k} = -\omega_k\bar{L}_k^0 + \sum_{l\neq j\in\mathscr{K}}\bar{\gamma}_{lj}\frac{\partial\mathscr{O}_{lj}}{\partial R_0^k} \tag{6}$$

and, therefore,

$$\bar{L}_k^0 = \sum_{l \neq j \in \mathscr{K}} \bar{\gamma}_{lj} \frac{1}{\omega_k} \frac{\partial \mathscr{O}_{lj}}{\partial R_0^k}. \tag{7}$$

Here we have used that $\langle \mathcal{L}_m | \mathrm{HF} \rangle = 0$ since $L_0^m = 0$ for $m > 0$ (as we are considering excited states here). Since the expression for $\bar{L}_k^0$ is the same in both the gradient and the coupling, substituting it for $\bar{\gamma}_{lj}$ leads to identical expressions.

For the cluster amplitudes, we have

$$\begin{aligned} \frac{\partial \mathscr{L}_n}{\partial t_\mu}\Big|_0 = 0 = L_\mu^m R_0^n + \langle L_m | \tau_\mu | R_n \rangle + \sum_\nu \bar{\zeta}_\nu A_{\nu\mu} + \sum_{k \in \mathscr{K}} \sum_{\nu\tau} \bar{L}_\nu^k P_{\nu\tau}^k \bar{F}(\boldsymbol{R}_k)_{\tau\mu} \\ + \sum_{l \neq j \in \mathscr{K}} \bar{\gamma}_{lj} \Big\{ \frac{\partial \mathscr{O}_{lj}}{\partial t_\mu} + \sum_{k \in \mathscr{K}} \frac{1}{\omega_k} \frac{\partial \mathscr{O}_{lj}}{\partial R_0^k} \Big(R_0^k \big(\eta_\mu - \sum_\nu L_\nu^k \bar{F}(\boldsymbol{R}_k)_{\nu\mu} \big) + \sum_\nu R_\nu^k F_{\nu\mu}^0 \Big) \Big\}. \end{aligned} \tag{8}$$

Note that the only terms that differ, with respect to the corresponding gradient expression, are the first two constant terms (that is, terms that do not depend on the multipliers).

Similarly, for the excited state amplitudes,

$$\begin{aligned} \frac{\partial \mathscr{L}_{mn}}{\partial R_\mu^l}\Big|_0 = 0 = L_\mu^m \delta_{ln} + \sum_{j \neq k \in \mathscr{K}} \zeta_{jk} \Big\langle \mathcal{L}_m \Big| \frac{\partial X_{jk}}{\partial R_\mu^l} \Big| \mathcal{R}_n \Big\rangle + \sum_\nu \bar{\zeta}_\nu Y_{\nu\mu}^l \\ + \sum_{p \in \mathscr{K}} \sum_\nu \bar{L}_\nu^p \Big\{ \delta_{pl} (A_{\nu\mu} - \omega_l \delta_{\nu\mu}) + \sum_\tau P_{\nu\tau}^p Y^l(\boldsymbol{R}_p)_{\tau\mu} \Big\} \\ + \sum_{j \neq k \in \mathscr{K}} \bar{\gamma}_{jk} \Big\{ \frac{\partial \mathscr{O}_{jk}}{\partial R_\mu^l} + \sum_{p \in \mathscr{K}} \frac{1}{\omega_p} \frac{\partial \mathscr{O}_{jk}}{\partial R_0^p} \Big(\delta_{pl} \eta_\mu + R_0^p \big(Y_\mu^{l,0} - \sum_\nu L_\nu^p Y^l(\boldsymbol{R}_p)_{\nu\mu} \big) + Y^{l,0}(\boldsymbol{R}_p)_\mu \Big) \Big\}. \end{aligned} \tag{9}$$

Also in this case, we find that the stationarity equation is identical to that obtained for the gradient, with the exception of the constant terms (here, these are the first two terms).

For ζ_{kl}, we similarly find

$$\begin{aligned} \frac{\partial \mathscr{L}_{mn}}{\partial \zeta_{kl}}\Big|_0 = 0 = \langle \mathcal{L}_m | X_{kl} | \mathcal{R}_n \rangle + \sum_\mu \bar{\zeta}_\mu z_\mu^{kl} + \sum_{j \in \mathscr{K}} \sum_{\mu\nu\tau} \bar{L}_\mu^j P_{\mu\tau}^j Z_{\tau\nu}^{kl} R_\nu^j \\ + \sum_{i \neq p \in \mathscr{K}} \bar{\gamma}_{ip} \Big\{ \sum_{j \in \mathscr{K}} \frac{1}{\omega_j} \frac{\partial \mathscr{O}_{ip}}{\partial R_0^j} \Big(R_0^j \big(z_{kl} - \sum_{\mu\nu} L_\mu^j Z_{\mu\nu}^{kl} R_\nu^j \big) + \tilde{z}_\mu^{kl} R_\mu^j \Big) \Big\}, \end{aligned} \tag{10}$$

where the only difference from the gradient is the first constant term.

Also the orbital response equation has modified constant term:

$$\frac{\partial \mathscr{L}_{mn}}{\partial \kappa_{ai}}\Big|_0 = 0 = \eta_{ai}^\kappa + \sum_{bj} \bar{\kappa}_{bj} A_{bjai}^{\mathrm{HF}}, \tag{11}$$

where

$$\eta_{ai}^{\kappa} = -\mathscr{P}_{ai} d_{ai}(\mathcal{L}_m, \mathcal{R}_n) + \sum_{k \in \mathscr{K}} \Big(\langle K_k \,|\, \bar{H}_{ai}^{\kappa} \,|\, R_k \rangle + \bar{L}_0^k \langle \mathrm{HF} \,|\, \bar{H}_{ai}^{\kappa} \,|\, \mathcal{R}_k \rangle \Big) + \langle \bar{\zeta} \,|\, \bar{H}_{ai}^{\kappa} \,|\, \mathrm{HF} \rangle. \qquad (12)$$

S2. ORTHOGONALITY CONDITIONS AND CHOICE OF METRIC

Below, we present some results where we use other choices of projection operator than $\mathscr{E}$ and $\mathscr{K}$. One of these will be referred to as "$\mathscr{E}$ with $T = 0$" since it can be obtained from natural metric $\mathscr{E}$ by ignoring the cluster amplitudes (i.e., by setting $T = 0$). This is the simplest metric, and it is the one we used in our thymine study.[1] The effect of setting $T = 0$ is simply to enforce orthogonality between the electronic states in the singles and doubles space, that is, to require that the configuration interaction part of the coupled cluster states are orthogonal.

The other additional metric we consider will be referred to as $\mathscr{K}_5$. This metric is obtained from $\mathscr{K}$ by not only projecting on the two states, but separately projecting onto the reference, singles, and doubles contributions from the two states. The subscript "5" signifies that this constitutes a projection onto a five-dimensional space instead of the two-dimensional space obtained using $\mathscr{K}$. In general, it appears that the choice of metric is often not important and will in most cases give similar results, although in some cases the "$\mathscr{E}$ with $T = 0$" metric provides a larger correction and a poorer description (see Section S10 in particular).

S3. ONE AND TWO-ELECTRON DENSITY CORRECTIONS

Here we list programmable expressions for the SCCSD corrections to the one and two-electron density matrices. We start with the one-electron matrix correction, where the only correction term is in the ia block:

$$\Delta d_{ia}(\mathcal{L},\mathcal{R}) = \zeta\langle L_2|[E_{ia}, X_3]|\mathrm{HF}\rangle R_0. \tag{13}$$

To evaluate this correction, we note that

$$\begin{aligned}\Delta d_{ia}(\mathcal{L},\mathcal{R}) &= \zeta\mathscr{P}^{-}_{ab}\langle L_2|[E_{ia}, R_1^a R_2^b]|\mathrm{HF}\rangle R_0\\ &= \frac{\zeta}{2}R_0\mathscr{P}^{-}_{ab}\Delta d^{ab}_{ia}(L_2),\end{aligned} \tag{14}$$

where

$$\Delta d^{ab}_{ia}(L_2) = \Delta d^{ab}_{ia}(L_2)^a + \Delta d^{ab}_{ia}(L_2)^b + \Delta d^{ab}_{ia}(L_2)^{d_1} + \Delta d^{ab}_{ia}(L_2)^{d_2} + \Delta d^{ab}_{ia}(L_2)^f, \tag{15}$$

with

$$\Delta d^{ab}_{ia}(L_2)^a = 4L_{bjck}R^a_{bj}R^b_{aick} \tag{16}$$

$$\Delta d^{ab}_{ia}(L_2)^b = -2L_{bjcl}R^a_{bj}R^b_{cial} \tag{17}$$

$$\Delta d^{ab}_{ia}(L_2)^{d_1} = -2L_{blck}R^a_{bi}R^b_{alck} \tag{18}$$

$$\Delta d^{ab}_{ia}(L_2)^{d_2} = -2L_{ckdj}R^a_{aj}R^b_{ckdi} \tag{19}$$

$$\Delta d^{ab}_{ia}(L_2)^f = 2L_{ckdl}R^a_{ai}R^b_{ckdl}. \tag{20}$$

In the two-electron density matrix, we need to evaluate the $ijka$, $bcka$, and $iajb$ blocks. Starting with the $ijka$ block, we have

$$d_{ijka}(\mathcal{L},\mathcal{R}) = \zeta\mathscr{P}^{-}_{ab}\langle L_2|[e_{ijka}, R_1^a R_2^b]|\mathrm{HF}\rangle R_0 \tag{21}$$

$$= \frac{\zeta}{2}R_0\mathscr{P}^{-}_{ab}\Delta d_{ijka}(L_2). \tag{22}$$

Splitting into subterms, we have

$$\begin{aligned}\Delta d_{ijka}(L_2) &= \Delta d_{ijka}(L_2)^a + \Delta d_{ijka}(L_2)^b + \Delta d_{ijka}(L_2)^{d_1} + \Delta d_{ijka}(L_2)^{d_{2,4}}\\ &\quad + \Delta d_{ijka}(L_2)^{d_3} + \Delta d_{ijka}(L_2)^{e_1} + \Delta d_{ijka}(L_2)^{e_2} + \Delta d_{ijka}(L_2)^{e_3}\\ &\quad + \Delta d_{ijka}(L_2)^g\end{aligned} \tag{23}$$

where, first, we have for the a term,

$$\Delta d_{ijka}(L_2)^a = 8\delta_{ij}Y_{ak} - 4\delta_{jk}Y_{ai} \tag{24}$$

with

$$Y_{ak} = X_{em}R^b_{akem}, \quad X_{em} = L_{emdl}R^a_{dl}. \tag{25}$$

For the b term, we have

$$\Delta d_{ijka}(L_2)^b = -4\delta_{ij}Y_{ak} + 2\delta_{jk}Y_{ai} - 4Z_{jiak} + 2Z_{jkai}, \tag{26}$$

where

$$Z_{jiak} = X_{ej}\tilde{R}^b_{eiak}, \quad \tilde{R}^k_{eiak} = R^k_{eiak}(1 + \delta_{ei,ak}) \tag{27}$$

The d_1 term reads

$$\Delta d_{ijka}(L_2)^{d_1} = -4\delta_{ij}\tilde{Y}_{ak} + 2\delta_{jk}Z_{ai}, \tag{28}$$

where

$$Z_{ai} = X_{ad}R^a_{di}, \quad X_{ad} = L_{dnem}R^b_{anem}, \tag{29}$$

and the $d_{2,4}$ term reads

$$\Delta d_{ijka}(L_2)^{d_{2,4}} = -4\delta ijW_{ak} + 2\delta_{jk}W_{ai} - 4X_{ji}R^a_{ak} + 2X_{jk}R^a_{ai}, \tag{30}$$

where

$$W_{ak} = X_{lk}R^a_{al}, \quad X_{lk} = L_{emfl}R^b_{emfk}. \tag{31}$$

For the d_3 term,

$$\Delta d_{ijka}(L_2)^{d_3} = -4Y_{ijak} + 2Y_{kjai}, \tag{32}$$

where

$$Y_{ijak} = X_{ijem}R^b_{emak}, \quad X_{ijem} = L_{djem}R^a_{di}. \tag{33}$$

Considering the e terms, we find for e_1 that

$$\Delta d_{ijka}(L_2)^{e_1} = 2Y_{ijka}, \quad Y_{ijka} = X_{ijen}R^b_{ekan}, \tag{34}$$

while for e_2, we have

$$\Delta d_{ijka}(L_2)^{e_2} = 2Y_{jkia}, \quad Y_{jkia} = X_{knej} R^b_{eian}, \tag{35}$$

and for e_3,

$$\Delta d_{ijka}(L_2)^{e_3} = 2Z_{kija}, \tag{36}$$

where

$$Z_{kija} = X_{kijl} R^a_{al}, \quad X_{kijl} = L_{elfj} R^b_{ekfi}. \tag{37}$$

The last term in the $ijka$ block, the g term, reads

$$\Delta d_{ijka}(L_2)^g = N(4\delta_{ij} R^a_{ak} - 2\delta_{jk} R^a_{ai}), \tag{38}$$

where

$$N = L_{emfn} R^b_{emfn}. \tag{39}$$

Next, we move on to the $abic$ block, where we can similarly write

$$\begin{aligned} \Delta d_{abic}(L_2) &= \Delta d_{abic}(L_2)^a + \Delta d_{abic}(L_2)^{b_1} + \Delta d_{abic}(L_2)^{b_2} + \Delta d_{abic}(L_2)^{c_1} \\ &+ \Delta d_{abic}(L_2)^{c_2} + \Delta d_{abic}(L_2)^{c_3}, \end{aligned} \tag{40}$$

with, for the a term,

$$\Delta d_{abic}(L_2)^a = 4Y_{ciba} - 2Y_{bica}, \quad Y_{ciba} = R^b_{cibm} X_{am}, \qquad X_{am} = L_{amck} R^a_{ck}. \tag{41}$$

For b_1, we have

$$\Delta d_{abic}(L_2)^{b_1} = 4Y_{ciab} - 2Y_{biac}, \quad Y_{ciab} = X_{cial} R^a_{bl}, \quad X_{cial} = R^b_{cidm} L_{dmal} \tag{42}$$

while for b_2, we have

$$\Delta d_{abic}(L_2)^{b_2} = 4R^a_{ci} X_{ba} - 2R^a_{bi} X_{ca}, \quad X_{ba} = R^b_{embn} L_{eman}. \tag{43}$$

For c_1, we get

$$\Delta d_{abic}(L_2)^{c_1} = -2Y_{cbia}, \quad Y_{cbia} = X_{iman} R^b_{cmbn}, \quad X_{iman} = R^a_{ci} L_{cman}. \tag{44}$$

For c_2,

$$\Delta d_{abic}(L_2)^{c_2} = -2Y_{ibac}, \quad Y_{ibac} = X_{ibal}R^a_{cl}, \quad X_{ibal} = R^b_{eibn}L_{elan} \tag{45}$$

and for c_3,

$$\Delta d_{abic}(L_2)^{c_3} = -2Y_{ciab}, \quad Y_{ciab} = X_{cial}R^a_{bl}, \quad X_{cial} = R^b_{cmfi}L_{fmal}. \tag{46}$$

Finally, we consider the $iajb$ block, where we first consider the term arising from the singles block L_1. For this contribution, we can write

$$\begin{aligned}\Delta d_{iajb}(L_1) = \mathscr{P}^{ab}_{ij}(&\Delta d_{iajb}(L_1)^a + \Delta d_{iajb}(L_1)^b \\ &+ \Delta d_{iajb}(L_1)^{c_1} + \Delta d_{iajb}(L_1)^{c_2} + \Delta d_{iajb}(L_1)^{c_3}),\end{aligned} \tag{47}$$

where, for the a and b terms, we have

$$\Delta d_{iajb}(L_1)^a = 2N(2R^b_{aibj} - R^b_{biaj}), \quad N = R^a_{ai}L_{ai} \tag{48}$$

and

$$\Delta d_{iajb}(L_1)^b = 8R^a_{ai}X_{bj} - 4R^a_{bi}X_{aj}, \quad X_{aj} = R^b_{ajdl}L_{dl}. \tag{49}$$

For c_1, we have

$$\Delta d_{iajb}(L_1)^{c_1} = -4Y_{aibj} + 2Y_{biaj}, \quad Y_{aibj} = R^b_{aibm}X_{mj}, \quad X_{mj} = L_{cm}R^a_{cj}. \tag{50}$$

For c_2,

$$\Delta d_{iajb}(L_1)^{c_2} = -4R^a_{ai}X_{jb} + 2R^a_{bi}X_{ja}, \quad X_{jb} = R^b_{djbm}L_{dm}, \tag{51}$$

and for c_3,

$$\Delta d_{iajb}(L_1)^{c_3} = -4Y_{bjai} + 2Y_{ajbi}, \quad Y_{bjai} = R^a_{bk}X_{kjai}, \quad X_{kjai} = L_{ek}R^b_{ejai}. \tag{52}$$

Next we consider the block in the $iajb$ block that stems from L_2. Here we have

$$\begin{aligned}\Delta d_{iajb}(L_2, R_1) = \mathscr{P}^{ab}_{ij}(&\Delta d_{iajb}(L_2, R_1)^a + \Delta d_{iajb}(L_2, R_1)^{b_1} \\ &+ \Delta d_{iajb}(L_2, R_1)^{b_{2a}} + \Delta d_{iajb}(L_2, R_1)^{b_{2b}} + \Delta d_{iajb}(L_2, R_1)^{b_{2c}} \\ &+ \Delta d_{iajb}(L_2, R_1)^{e_1} + \Delta d_{iajb}(L_2, R_1)^{e_2} + \Delta d_{iajb}(L_2, R_1)^{e_3} \\ &+ \Delta d_{iajb}(L_2, R_1)^{f_1} + \Delta d_{iajb}(L_2, R_1)^{f_2} + \Delta d_{iajb}(L_2, R_1)^{g}).\end{aligned} \tag{53}$$

For the a term, we have

$$\Delta d_{iajb}(L_2,R_1)^{a} = 8Y_{ai}R_{bj} - 4Y_{bi}R_{aj}, \quad Y_{ai} = R^{a}_{ck}R^{b}_{dlai}L_{ckdl}. \tag{54}$$

For the b_1 term,

$$\Delta d_{iajb}(L_2,R_1)^{b_1} = N(4R^{b}_{aibj} - 2R^{b}_{ajbi}), \quad N = R^{a}_{ck}L_{ckfn}R_{fn}. \tag{55}$$

For b_{2a},

$$\begin{aligned}\Delta d_{iajb}(L_2,R_1)^{b_{2a}} = -4Z_{bjai} + 2Z_{ajbi}, \quad Z_{bjai} = Y_{bd}R^{b}_{djai},\\ Y_{bd} = R_{bn}X_{dn}, \quad X_{dn} = L_{dnck}R^{a}_{ck}.\end{aligned} \tag{56}$$

For b_{2b},

$$\begin{aligned}\Delta d_{iajb}(L_2,R_1)^{b_{2b}} = -4Z_{aibj} + 2Z_{biaj}, \quad Z_{aibj} = R^{b}_{aibl}Y_{lj},\\ Y_{lj} = X_{fl}R_{fj}, \quad X_{fl} = L_{flck}R^{a}_{ck}.\end{aligned} \tag{57}$$

For b_{2c},

$$\Delta d_{iajb}(L_2,R_1)^{b_{2c}} = -4R_{ai}Y_{bj} + 2R_{bi}Y_{aj}, \quad Y_{bj} = R^{b}_{djbm}X_{dm}, \quad X_{dm} = L_{dmck}R^{a}_{ck}. \tag{58}$$

For e_1, we have three subterms,

$$\Delta d_{iajb}(L_2,R_1)^{e_1} = \Delta d_{iajb}(L_2,R_1)^{e_{1a}} + \Delta d_{iajb}(L_2,R_1)^{e_{1b}} + \Delta d_{iajb}(L_2,R_1)^{e_{1c}}, \tag{59}$$

where

$$\Delta d_{iajb}(L_2,R_1)^{e_{1a}} = -4Z_{aibj} + 2Z_{biaj}, \quad Z_{aibj} = R^{b}_{aibm}Y_{mj}, \quad Y_{mj} = X_{cm}R^{a}_{cj} \tag{60}$$

$$\Delta d_{iajb}(L_2,R_1)^{e_{1b}} = -4R^{a}_{ai}Y_{bj} + 2R^{a}_{bi}Y_{aj}, \quad Y_{bj} = R^{b}_{djbm}X_{dm} \tag{61}$$

$$\Delta d_{iajb}(L_2,R_1)^{e_{1c}} = -4Z_{bjai} + 2Z_{ajbi}, \quad Z_{bjai} = Y_{be}R^{b}_{ejai}, \quad Y_{be} = R^{a}_{bk}X_{ek}, \tag{62}$$

with

$$X_{ai} = L_{aibj}R_{bj}. \tag{63}$$

For e_2, we have

$$\Delta d_{iajb}(L_2,R_1)^{e_2} = \Delta d_{iajb}(L_2,R_1)^{e_{2a}} + \Delta d_{iajb}(L_2,R_1)^{e_{2b}} + \Delta d_{iajb}(L_2,R_1)^{e_{2c}}, \tag{64}$$

where

$$\Delta d_{iajb}(L_2,R_1)^{e_{2a}} = 2Z_{aijb}, \quad Z_{aijb} = Y_{aijn}R_{bn}, \quad Y_{aijn} = R^b_{alei}X_{jnel} \tag{65}$$

$$\Delta d_{iajb}(L_2,R_1)^{e_{2c}} = 2Z_{ajib}, \quad Z_{ajib} = Y_{ajin}R_{bn}, \quad Y_{ajin} = R^b_{alej}X_{inel}, \tag{66}$$

with

$$X_{inel} = R^a_{ic}L_{cnel}, \tag{67}$$

and

$$\begin{aligned}\Delta d_{iajb}(L_2,R_1)^{e_{2b}} = 2Z_{ijba}, \quad Z_{ijba} = Y_{ijkb}R^a_{ak},\\ Y_{ijkb} = X_{ijkn}R_{bn}, \quad X_{ijkn} = R^b_{djei}L_{ekdn}.\end{aligned} \tag{68}$$

Similarly, for e_3,

$$\Delta d_{iajb}(L_2,R_1)^{e_3} = \Delta d_{iajb}(L_2,R_1)^{e_{3a}} + \Delta d_{iajb}(L_2,R_1)^{e_{3b}} + \Delta d_{iajb}(L_2,R_1)^{e_{3c}}, \tag{69}$$

where

$$\Delta d_{iajb}(L_2,R_1)^{e_{3a}} = 2Z_{abij}, \quad Z_{abij} = R^b_{albm}Y_{lmij}, \quad Y_{lmij} = X_{ilcm}R^a_{cj} \tag{70}$$

$$\Delta d_{iajb}(L_2,R_1)^{e_{3b}} = 2Z_{aibj}, \quad Z_{aibj} = Y_{aidm}R^b_{djbm}, \quad Y_{aidm} = R^a_{ak}X_{ikdm} \tag{71}$$

$$\Delta d_{iajb}(L_2,R_1)^{e_{3c}} = 2Z_{ajib}, \quad Z_{ajib} = Y_{ajik}R^a_{bk}, \quad Y_{ajik} = R^b_{alej}X_{ilek}, \tag{72}$$

with

$$X_{ilek} = R_{ci}L_{clek}. \tag{73}$$

For f_1, we have

$$\Delta d_{iajb}(L_2,R_1)^{f_1} = 8Y_{ai}R^a_{bj} - 4Y_{bi}R^a_{aj}, \quad Y_{ai} = R^b_{aidl}X_{dl}, \quad X_{dl} = R_{fn}L_{dlfn}. \tag{74}$$

For f_2, we have

$$\begin{aligned}\Delta d_{iajb}(L_2,R_1)^{f_2} = \mathscr{P}(R_1,R^a_1)(\Delta d_{iajb}(L_2,R_1)^{f_{2a}}\\ + \Delta d_{iajb}(L_2,R_1)^{f_{2b}} + \Delta d_{iajb}(L_2,R_1)^{f_{2c}})\end{aligned} \tag{75}$$

with

$$\Delta d_{iajb}(L_2,R_1)^{f_{2a}} = -4R^a_{ai}Y_{bj} + 2R^a_{bi}Y_{aj}, \quad Y_{bj} = R_{bn}X_{jn}, \quad X_{jn} = R^b_{dleij}L_{dlen} \tag{76}$$

$$\begin{aligned}\Delta d_{iajb}(L_2,R_1)^{f_{2b}} = -4Z_{aijb} + 2Z_{bija}, \quad Z_{aijb} = Y_{aijn}R_{bn},\\ Y_{aijn} = R^b_{aidl}X_{jndl}, \quad X_{jndl} = R^a_{cj}L_{cndl}\end{aligned} \tag{77}$$

$$\Delta d_{iajb}(L_2,R_1)^{f_{2c}} = -4R_{ai}Y_{bj} + 2R_{bi}Y_{aj}, \quad Y_{bj} = X_{bc}R^a_{cj}, \quad X_{bc} = R^b_{bmdl}L_{cmdl}. \tag{78}$$

Finally, for the g term, we have

$$\Delta d_{iajb}(L_2, R_1)^g = N(4R^a_{ai}R_{bj} - 2R^a_{bi}R_{aj}), \quad N = R^b_{dlem}L_{dlem}. \tag{79}$$

Although the terms above give the SCCSD corrections to densities relative to CCSD, there are also some additional Hartree-Fock level density terms that enter into the nuclear derivatives. These terms arise because of the reference term in the overlap (R^k_0), which produces a reference contributions to the effective SCCSD density matrices. In particular, the Lagrangians have the term

$$\bar{L}^k_0 \langle \mathrm{HF} | \bar{H} | \mathcal{R}_k \rangle \tag{80}$$

which implies density contributions of the form

$$\langle \mathrm{HF} | E_{pq} | \mathrm{HF} \rangle \tag{81}$$

$$\langle \mathrm{HF} | e_{pqrs} | \theta \rangle, \quad |\theta\rangle = \sum_\mu \theta_\mu |\mu\rangle. \tag{82}$$

These Hartree-Fock density contributions are well-known and we refer to the literature for programmable expressions.[2]

S4. RESPONSE VECTORS FOR EXCITED STATE STATIONARITY

Let us start with

$$\rho_\mu^m = \langle\bar{\zeta}|Y_\mu^m|\mathrm{HF}\rangle. \tag{83}$$

We have

$$\begin{aligned}
\rho_{ai}^m &= \zeta\langle\bar{\zeta}|[\hat{H}, E_{ai}R_2^n]|\mathrm{HF}\rangle \\
&= \frac{1}{2}\zeta\bar{R}_{bjck}^n\langle\bar{\zeta}|[H, E_{ai}E_{bj}E_{ck}|\mathrm{HF}\rangle \\
&= \frac{1}{2}\zeta\bar{R}_{bjck}^n\omega_{ijk}^{abc},
\end{aligned} \tag{84}$$

where

$$\omega_{ijk}^{abc} = \sum_{\alpha=1}^{7} {}^{\alpha}\omega_{ijk}^{abc} \tag{85}$$

with

$$\begin{aligned}
{}^1\omega_{ijk}^{abc} &= 2\bar{\zeta}_{aibj}F_{kc} \\
{}^2\omega_{ijk}^{abc} &= 2\bar{\zeta}_{ai}L_{jbkc} \\
&\quad - P_{jk}^{bc}(F_{jc}\bar{\zeta}_{aibk} + L_{jlkc}\bar{\zeta}_{aibl} - L_{dbkc}\bar{\zeta}_{aidj}) \\
{}^3\omega_{ijk}^{abc} &= 2\bar{\zeta}_{aick}F_{jb} \\
{}^4\omega_{ijk}^{abc} &= 2\bar{\zeta}_{bj}L_{iakc} \\
&\quad - P_{ik}^{ac}(F_{ic}\bar{\zeta}_{bjak} + L_{ilkc}\bar{\zeta}_{bjal} - L_{dakc}\bar{\zeta}_{bjdi}) \\
{}^5\omega_{ijk}^{abc} &= P_{ijk}^{abc}(-L_{jbic}\bar{\zeta}_{ak} + g_{iljc}\bar{\zeta}_{albk} - g_{ibdc}\bar{\zeta}_{djak}) \\
{}^6\omega_{ijk}^{abc} &= 2\bar{\zeta}_{ck}L_{iajb} \\
&\quad - P_{ij}^{ab}(F_{ib}\bar{\zeta}_{ckaj} + L_{iljb}\bar{\zeta}_{ckal} - L_{dajb}\bar{\zeta}_{ckdi}) \\
{}^7\omega_{ijk}^{abc} &= 2\bar{\zeta}_{bjck}F_{ia}.
\end{aligned} \tag{86}$$

Similarly,

$$\begin{aligned}
\rho_{aibj}^m &= -\zeta\langle\bar{\zeta}|[\hat{H}, E_{ai}E_{bj}R_1^n]|\mathrm{HF}\rangle \\
&= -\zeta R_{ck}^n\omega_{ijk}^{abc},
\end{aligned} \tag{87}$$

so that we may reuse the formula for ω_{ijk}^{abc}. Note that, in order to avoid N^7 scaling, we do not implement ω_{ijk}^{abc} but rather its partial contractions with R_{ck}^n and R_{bjck}^n.

Next we consider

$$\sigma_\mu^m = \langle \Lambda | [Y_\mu^m, R] | \text{HF} \rangle. \tag{88}$$

Here we also define a useful tensor:

$$\begin{aligned}\sigma_{ai}^m &= \zeta \langle \Lambda | [[\hat{H}, E_{ai} R_2^n], R_1] | \text{HF} \rangle \\ &= \frac{\zeta}{2} \bar{R}_{bjck}^n R_{dl} \langle \Lambda | [[\hat{H}, E_{ai} E_{bj} E_{ck}], E_{dl}] | \text{HF} \rangle \\ &= \frac{\zeta}{2} \bar{R}_{bjck}^n R_{dl} \gamma_{ijkl}^{abcd}.\end{aligned} \tag{89}$$

This tensor also has 7 terms,

$$\gamma_{ijkl}^{abcd} = \sum_{\alpha=1}^{7} {}^{\alpha}\gamma_{ijkl}^{abcd}, \tag{90}$$

with

$${}^{1}\gamma_{ijkl}^{abcd} = 2\Lambda_{aibj} L_{kcld} \tag{91}$$

$${}^{2}\gamma_{ijkl}^{abcd} = -P_{jkl}^{bcd} L_{kcjd} \Lambda_{aibl} \tag{92}$$

$${}^{3}\gamma_{ijkl}^{abcd} = 2\Lambda_{aick} L_{jbld} \tag{93}$$

$${}^{4}\gamma_{ijkl}^{abcd} = -P_{ikl}^{acd} L_{kcid} \Lambda_{bjal} \tag{94}$$

$${}^{5}\gamma_{ijkl}^{abcd} = P_{ijk}^{abc} (g_{kbid} \Lambda_{cjal} + g_{lbic} \Lambda_{djak}) \tag{95}$$

$${}^{6}\gamma_{ijkl}^{abcd} = -P_{jil}^{bad} L_{iajd} \Lambda_{ckbl} \tag{96}$$

$${}^{7}\gamma_{ijkl}^{abcd} = 2\Lambda_{bjck} L_{iald}. \tag{97}$$

Again, we obtain expressions by using that

$$\begin{aligned}\sigma_{aibj}^m &= -\zeta \langle \Lambda | [[H, E_{ai} E_{bj} R_1^n], R_1] | \text{HF} \rangle \\ &= -\zeta R_{ck}^n R_{dl} \gamma_{ijkl}^{abcd}.\end{aligned} \tag{98}$$

It is useful to explicitly denote the dependence of these vectors, i.e. by writing

$$\rho_\mu^m = \rho_\mu^m(\bar{\zeta}) \tag{99}$$

$$\sigma_\mu^m = \sigma_\mu^m(\Lambda, R). \tag{100}$$

In particular, this allows to see that

$$R_\mu^m \rho_\mu^m(\bar{\zeta}) = \zeta \langle \bar{\zeta} | [\hat{H}, X_3] | \text{HF} \rangle \tag{101}$$

$$R_\mu^m \sigma_\mu^m(\Lambda, R) = \zeta \langle \Lambda | [[\hat{H}, X_3], R] | \text{HF} \rangle \tag{102}$$

and hence we can calculate the ζ-derivative terms (in $\partial \mathscr{L}_n / \partial \zeta$) by reusing the code from the state derivatives and then evaluate the response by evaluating the dot products in Eqs. (101) and (102), appropriately premultiplying by ζ^{-1}.

S5. RESPONSE CONTRIBUTIONS FROM THE ORTHOGONALITY

When differentiating the orthogonality condition, we can in most cases reuse expressions implemented in the original SCCSD paper.[3] The single exception is the t_μ derivative, which gives rise to terms of the form

$$\langle\mathcal{L}|\tau_\mu \exp(T)|\mathcal{R}\rangle = L_\mu R_0 + \sum_\nu \langle\mathcal{L}|\tau_\mu|\nu\rangle\langle\nu|\exp(T)|\mathcal{R}\rangle \tag{103}$$

$$= L_\mu R_0 + \langle L|\tau_\mu|q\rangle R_0 + \langle L|\tau_\mu|QR\rangle \tag{104}$$

$$= L_\mu R_0 + J_\mu(L,q)R_0 + J_\mu(L,QR), \tag{105}$$

where

$$|q\rangle = \sum_\mu q_\mu|\mu\rangle, \quad q_\mu = \langle\mu|\exp(T)|\mathrm{HF}\rangle \tag{106}$$

and

$$|QR\rangle = \sum_\mu (\boldsymbol{QR})_\mu|\mu\rangle, \quad Q_{\mu\nu} = \langle\mu|\exp(T)|\nu\rangle. \tag{107}$$

Programmable expressions for $\boldsymbol{J}$ are given in Ref. 4 and for $\boldsymbol{q}$ and $\boldsymbol{Q}$, as well as the terms needed to evaluate the relevant vectors $\langle\mathcal{L}|$ and $|\mathcal{R}\rangle$, we refer to Ref. 3.

S6. COMPARISON WITH NUMERICAL GRADIENTS

To test the correctness of the analytical gradient implementation, we compare analytical and numerical gradients for a system (HOF–He) with no point group symmetry, see Tables S1 and S2. For all the SCCSD metrics, we find deviations of $\leq 10^{-9}$ using a five-point stencil ($h = 5 \cdot 10^{-4}$) to evaluate the numerical gradients.

TABLE S1. Comparison of numerical and analytical gradients (I). The gradients are for the fourth singlet excited state with SCCSD/cc-pVDZ applied to states 3 and 4. Numerical gradients were evaluated with a five-point stencil using a displacement of $h = 5 \cdot 10^{-4}$. All values are in atomic units.

	Molecular geometry		
O	0.000 000 000	0.000 000 000	0.000 000 000
H	2.000 000 000	0.000 000 000	0.000 000 000
F	0.000 000 000	2.500 000 000	0.000 000 000
He	0.000 000 000	5.000 000 000	5.000 000 000
	Analytical gradient SCCSD($\mathscr{E}$)		
O	0.113 550 411	0.044 530 280	0.000 003 607
H	−0.113 650 917	0.000 137 583	0.000 023 901
F	0.000 090 945	−0.044 634 812	0.000 011 086
He	0.000 009 560	−0.000 033 051	−0.000 038 594
	Numerical gradient SCCSD($\mathscr{E}$)		
O	0.113 550 412	0.044 530 279	0.000 003 607
H	−0.113 650 917	0.000 137 583	0.000 023 901
F	0.000 090 946	−0.044 634 813	0.000 011 087
He	0.000 009 560	−0.000 033 051	−0.000 038 593
	Analytical gradient SCCSD($\mathscr{K}$)		
O	0.113 553 037	0.044 515 419	0.000 003 607
H	−0.113 655 685	0.000 140 260	0.000 023 901
F	0.000 093 087	−0.044 622 629	0.000 011 085
He	0.000 009 560	−0.000 033 051	−0.000 038 594
	Numerical gradient SCCSD($\mathscr{K}$)		
O	0.113 553 037	0.044 515 419	0.000 003 607
H	−0.113 655 684	0.000 140 260	0.000 023 901
F	0.000 093 087	−0.044 622 628	0.000 011 086
He	0.000 009 559	−0.000 033 051	−0.000 038 593

TABLE S2. Comparison of numerical and analytical gradients (II). The gradients are for the fourth singlet excited state with SCCSD/cc-pVDZ applied to states 3 and 4. Numerical gradients were evaluated with a five-point stencil using a displacement of $h = 5 \cdot 10^{-4}$. All values are in atomic units.

	Molecular geometry		
O	0.000 000 000	0.000 000 000	0.000 000 000
H	2.000 000 000	0.000 000 000	0.000 000 000
F	0.000 000 000	2.500 000 000	0.000 000 000
He	0.000 000 000	5.000 000 000	5.000 000 000
	Analytical gradient SCCSD($\mathscr{K}_5$)		
O	0.113 554 289	0.044 515 386	0.000 003 607
H	−0.113 655 265	0.000 138 170	0.000 023 901
F	0.000 091 415	−0.044 620 504	0.000 011 086
He	0.000 009 561	−0.000 033 052	−0.000 038 595
	Numerical gradient SCCSD($\mathscr{K}_5$)		
O	0.113 554 289	0.044 515 385	0.000 003 607
H	−0.113 655 264	0.000 138 170	0.000 023 901
F	0.000 091 414	−0.044 620 503	0.000 011 087
He	0.000 009 561	−0.000 033 052	−0.000 038 595
	Analytical gradient SCCSD($\mathscr{E}$ with $T = 0$)		
O	0.113 604 811	0.044 561 109	0.000 003 606
H	−0.113 649 517	0.000 067 838	0.000 023 912
F	0.000 035 140	−0.044 595 872	0.000 011 113
He	0.000 009 565	−0.000 033 075	−0.000 038 631
	Numerical gradient SCCSD($\mathscr{E}$ with $T = 0$)		
O	0.113 604 811	0.044 561 109	0.000 003 605
H	−0.113 649 516	0.000 067 838	0.000 023 913
F	0.000 035 141	−0.044 595 872	0.000 011 113
He	0.000 009 566	−0.000 033 075	−0.000 038 631

TABLE S3. The gradients are for the fourth singlet excited state with CCSD/cc-pVDZ. All values are in atomic units.

	Molecular geometry		
O	0.000 000 000	0.000 000 000	0.000 000 000
H	2.000 000 000	0.000 000 000	0.000 000 000
F	0.000 000 000	2.500 000 000	0.000 000 000
He	0.000 000 000	5.000 000 000	5.000 000 000
	Analytical gradient CCSD		
O	0.114 082 520	0.044 666 863	0.000 003 607
H	−0.113 857 682	−0.000 269 094	0.000 023 908
F	−0.000 234 401	−0.044 364 645	0.000 011 217
He	0.000 009 563	−0.000 033 124	−0.000 038 732

S7. FORMALDIMINE MECIS

Minimum energy conical intersections for protonated formaldimine are given in Table S4. MECI geometries, along with $\boldsymbol{g}$ and $\boldsymbol{h}$ for SCCSD($\mathscr{E}$), can be found in the Zenodo repository associated with the manuscript.[5]

	CCSD	SCCSD				XMS-CASPT2[6]	MP2/ADC(2)[6]	TDDFT[6]
		($\mathscr{S}$)	($\mathscr{E}$)	($\mathscr{K}$)	($\mathscr{K}_5$)			
S_0 minimum	1.271	-	-	-	-	1.281	1.275	1.274
Planar MECI	1.426	1.426	1.426	1.426	1.426	1.420	1.389	1.541
Distorted MECI	1.433	1.433	1.433	1.433	1.433	-	-	-

TABLE S4. C-N bond lengths (in Å) in protonated formaldimine at the S_0 minimum and at S_2/S_1 minimum energy conical intersections. The TD-DFT calculations (from Ref. 6) use the cc-pVDZ basis set. All other calculations are with the cc-pVTZ basis set.

S8. MULTIPLE SOLUTIONS

A. Protonated formaldimine

In Figure S1, we present an interpolation from the Franck-Condon point to the distorted MECI of protonated formaldimine obtained by using SCCSD($\mathscr{E}$). The upper panel shows the potential energies of the two excited states involved as a function of the interpolation coordinate x; the bottom panel shows the value of ζ over the same range. A discontinuity in the ζ value is visible at $x \approx 0.42$, though this is not visible in the potential energy curves. In Figure S2 we present a scan of the overlap condition versus ζ at $x \approx 0.42$, revealing that the discontinuity is caused by a change of solution in the overlap condition.

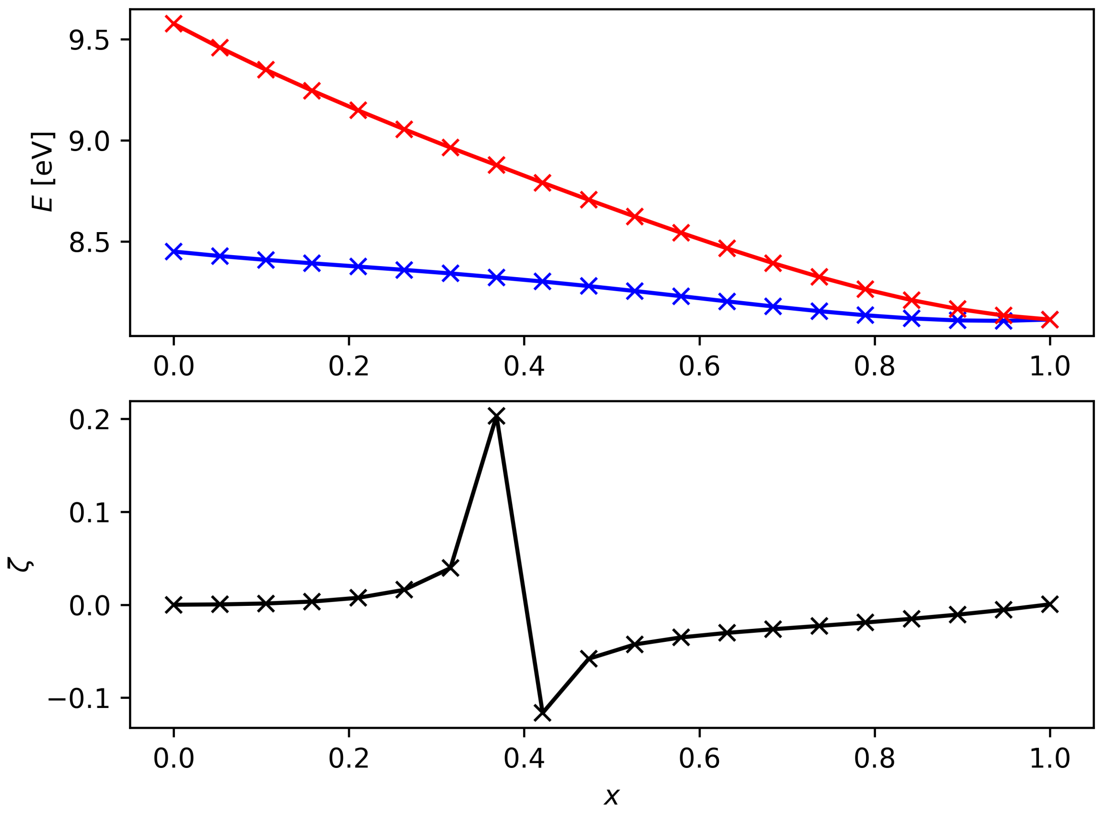

FIG. S1. Interpolation from Franck-Condon geometry ($x = 0.0$) to distorted MECI ($x = 1.0$) using SCCSD($\mathscr{E}$). At around $x \approx 0.42$, one solution of the orthogonality condition is replaced by another, as can be seen from the discontinuity in the ζ value in the lower panel.

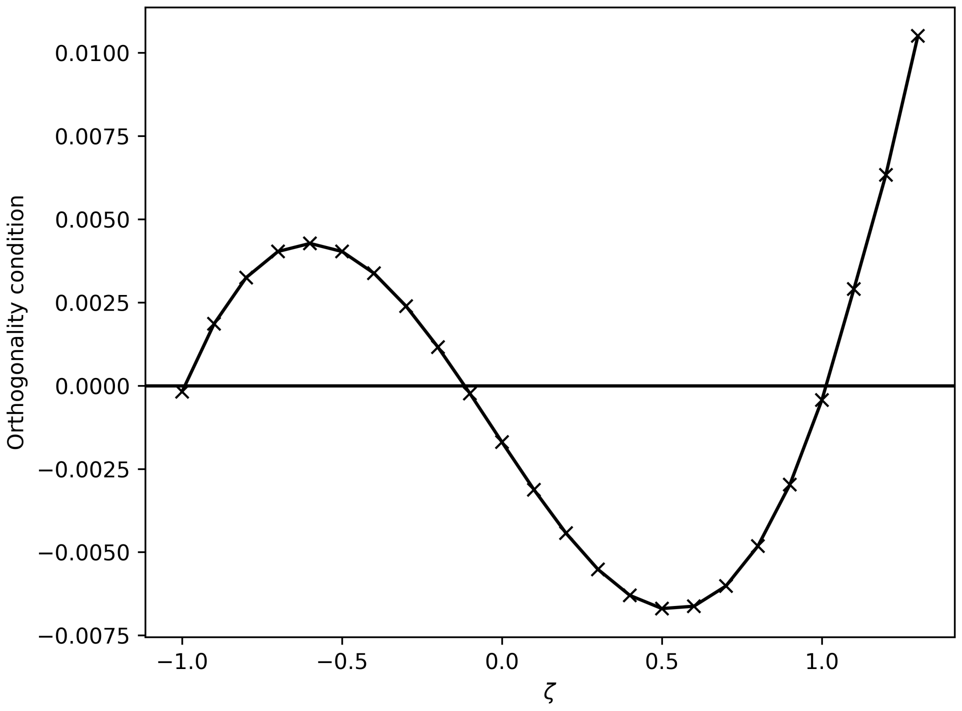

FIG. S2. The orthogonality condition as a function of ζ at the point ($x \approx 0.42$) where we see a discontinuity in the interpolation from the Franck-Condon geometry to the distorted MECI using SCCSD($\mathscr{E}$). The solution at $\zeta \approx -0.1$ is different from the solution at around $\zeta \approx 1.0$, where the latter corresponds to the solution that was obtained for the previous x-value ($x \approx 0.37$).

B. Lithium hydride

Figure S3 shows the overlap condition as a function of ζ at a bond length of 4.4 bohr using SCCSD($\mathscr{E}$), where the description given by the method is already poor compared to CCSD. Here, we can see that there are two solutions, and the one giving the larger correction (at around $\zeta \approx 20$) is the one obtained in the scan shown in the main text. The other solution becomes well-behaved at bond lengths longer than 4.4 bohr.

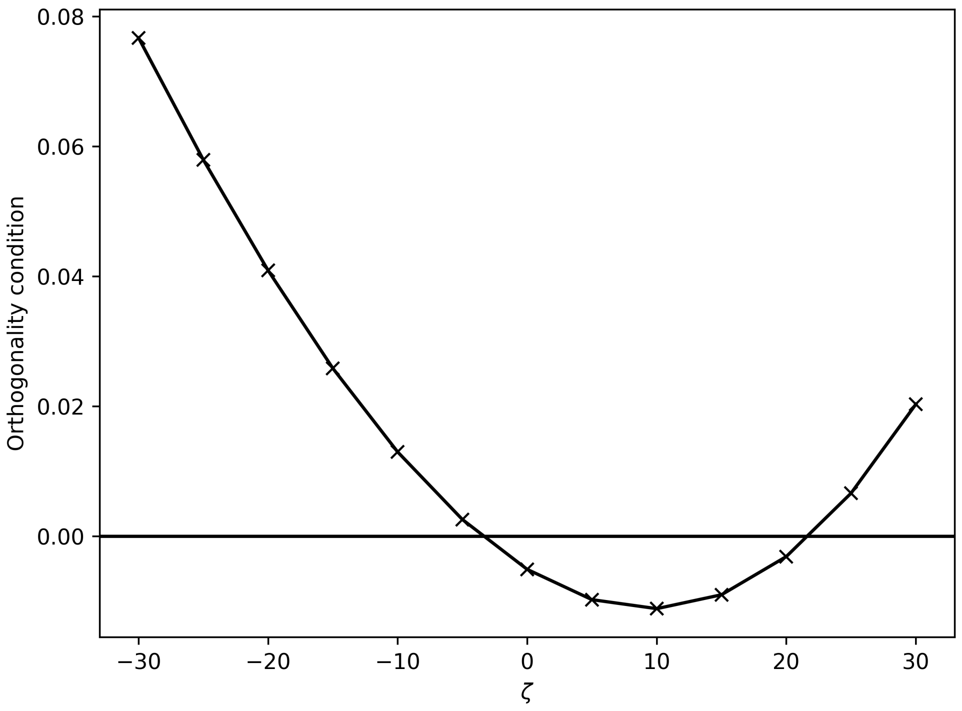

FIG. S3. The orthogonality condition as a function of ζ for LiH and SCCSD($\mathscr{E}$) at a bond distance of 4.4 bohr, where the method is already deviating significantly from CCSD. The solution at $\zeta \approx -3$ is different from the solution at around $\zeta \approx 20$, where the latter corresponds to the solution that is obtained in the scan shown in the main text. The solution at $\zeta \approx -3$ becomes well-behaved for larger bond lengths, unlike the solution at $\zeta \approx 20$.

S9. THYMINE MECIS

Minimum energy conical intersections for thymine are given in Table S5. MECI geometries can be found in the Zenodo repository associated with the manuscript.[5]

	S_0 minimum	Distorted MECI				
	CCSD	CCSD	SCCSD			
			$(\mathscr{S})$	$(\mathscr{E})$	$(\mathscr{K})$	$(\mathscr{K}_5)$
C_4-O_8 (Å)	1.224	1.265*	1.265	1.265	1.265	1.265
C_5-C_6 (Å)	1.357	1.446*	1.446	1.446	1.446	1.446

TABLE S5. C_4-O_8 and C_5-C_6 bond lengths (in Å) for thymine at the S_2/S_1 minimum energy conical intersections. The S_0 minimum was optimized using the aug-cc-pVDZ basis. All other calculations use the cc-pVDZ basis set. The asterisk indicates that the geometry was converged only to within 10^{-3} a.u. in the gradient. Other geometries are converged to within 10^{-4} a.u.

S10. COUPLING ELEMENTS FOR LITHIUM HYDRIDE

Coupling elements for the $2\,^1\Sigma^+/3\,^1\Sigma^+$ and $3\,^1\Sigma^+/4\,^1\Sigma^+$ states are shown in Figure S4.

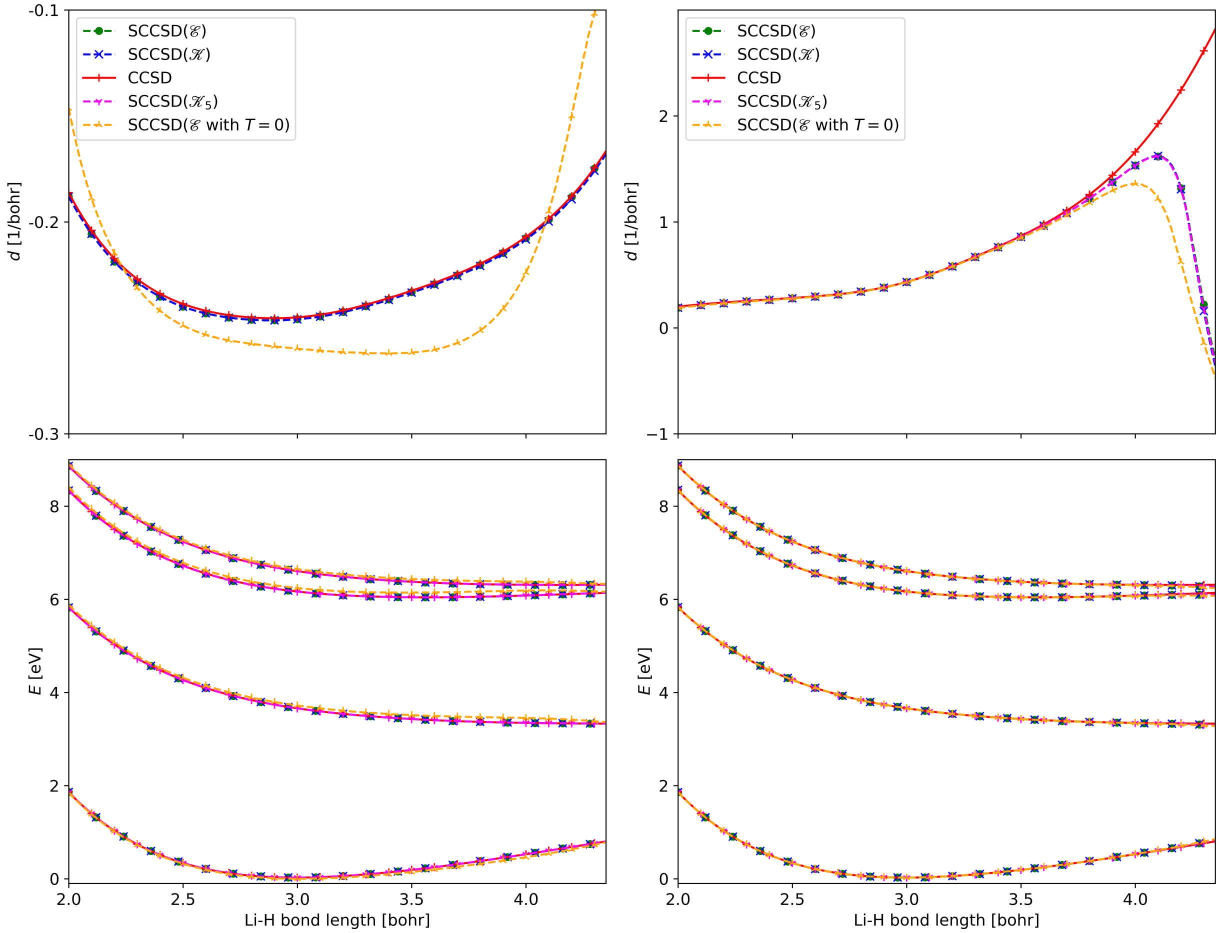

FIG. S4. Coupling elements between the $2\,^1\Sigma^+/3\,^1\Sigma^+$ (top left) and $3\,^1\Sigma^+/4\,^1\Sigma^+$ (top right) states. The magnitude of the coupling is calculated as $d = 2(d_{\mathrm{Li}} - d_{\mathrm{H}})$. Energies of the considered states ($1\,^1\Sigma^+$, $2\,^1\Sigma^+$, $3\,^1\Sigma^+$, $4\,^1\Sigma^+$) are given in the bottom left and right panels. The couplings are the right coupling elements, that is, the nuclear derivative acts on the ket vectors (in this case, $3\,^1\Sigma^+$ and $4\,^1\Sigma^+$ for the left and right panels, respectively).

REFERENCES

[1]E. F. Kjønstad, O. J. Fajen, A. C. Paul, S. Angelico, D. Mayer, M. Gühr, T. J. A. Wolf, T. J. Martínez, and H. Koch, "Unexpected hydrogen dissociation in thymine: predictions from a novel coupled cluster theory," arXiv , 10.48550/arXiv.2403.01045 (2024).

[2]T. Helgaker, P. Jorgensen, and J. Olsen, *Molecular electronic-structure theory* (John Wiley & Sons, 2013).

[3]E. F. Kjønstad and H. Koch, "An orbital invariant similarity constrained coupled cluster model," J. Chem. Theory Comput. **15**, 5386–5397 (2019).

[4]E. F. Kjønstad and H. Koch, "Communication: Non-adiabatic derivative coupling elements for the coupled cluster singles and doubles model," J. Chem. Phys. **158**, 161106 (2023).

[5]E. F. Kjønstad, S. Angelico, and H. Koch, "Data for "Coupled cluster theory for nonadiabatic dynamics: nuclear gradients and nonadiabatic couplings in similarity constrained coupled cluster theory"," 10.5281/zenodo.11122519 (2024).

[6]J. T. Taylor, D. J. Tozer, and B. F. E. Curchod, "On the description of conical intersections between excited electronic states with LR-TDDFT and ADC(2)," J. Chem. Phys. **159**, 214115 (2023).
